# Activation of GATA4 gene expression at the early stage of cardiac specification

**DOI:** 10.3389/fchem.2014.00012

**Published:** 2014-03-14

**Authors:** Ayse E. Yilbas, Alison Hamilton, Yingjian Wang, Hymn Mach, Natascha Lacroix, Darryl R. Davis, Jihong Chen, Qiao Li

**Affiliations:** ^1^Department of Cellular and Molecular Medicine, University of OttawaOttawa, ON, Canada; ^2^Department of Pathology and Laboratory Medicine, University of OttawaOttawa, ON, Canada; ^3^Faculty of Medicine, University of Ottawa Heart Institute, University of OttawaOttawa, ON, Canada

**Keywords:** GATA4, gene regulation, histone acetylation, cardiac differentiation, cardiomyocytes

## Abstract

Currently, there are no effective treatments to directly repair damaged heart tissue after cardiac injury since existing therapies focus on rescuing or preserving reversibly damaged tissue. Cell-based therapies using cardiomyocytes generated from stem cells present a promising therapeutic approach to directly replace damaged myocardium with new healthy tissue. However, the molecular mechanisms underlying the commitment of stem cells into cardiomyocytes are not fully understood and will be critical to guide this new technology into the clinic. Since GATA4 is a critical regulator of cardiac differentiation, we examined the molecular basis underlying the early activation of GATA4 gene expression during cardiac differentiation of pluripotent stem cells. Our studies demonstrate the direct involvement of histone acetylation and transcriptional coactivator p300 in the regulation of GATA4 gene expression. More importantly, we show that histone acetyltransferase (HAT) activity is important for GATA4 gene expression with the use of curcumin, a HAT inhibitor. In addition, the widely used histone deacetylase inhibitor valproic acid enhances both histone acetylation and cardiac specification.

## Introduction

Cardiovascular diseases are among the leading causes of death for men and women worldwide with end stage heart failure being the primary cause of mortality and morbidity (Go et al., 2013). Most survivors of cardiac damage live with inadequate heart function which reflects the limited ability of human cardiac cells to proliferate and replace injured myocardium. For these patients, treatment with multi or pluripotent stem cells represents a promising renewable therapy since these cells can be generally programmed to replace damaged tissue. Thus, elucidating the molecular mechanisms underlying gene cascades to commit the stem cells into a cardiac fate is essential for developing the best strategies to direct cardiac differentiation.

The initiation of cardiac differentiation is indicated by the expression of the early cardiac genes, which include GATA binding protein 4 (GATA4), myocyte enhancer factor 2C (MEF2C) and NK2 transcription factor related locus 5 (Nkx2.5). GATA4 is a key regulator of endoderm and mesoderm development in the postgastrula embryo (Rossi et al., 2001). It also controls the expression of genes critical for cardiomyogenesis while self-regulating to eventually suppress its own expression as well (Rossi et al., 2001). Over-expression of GATA4 in pluripotent P19 stem cells or mouse embryonic stem (ES) cells can drive pluripotent cells to a cardiac fate by increasing cardiac differentiation under conditions known to favor a cardiac lineage (Grepin et al., 1997).

On a molecular level, GATA4 interacts with many transcription factors to regulate the differentiation of progenitor cells. MEF2C is a cofactor of GATA4 and plays a role in the development of cardiac, skeletal, and smooth muscle (Morin et al., 2000). In addition, GATA4 not only regulates the expression of Nkx2.5 by enhancing activation (Akazawa and Komuro, 2005), it also binds to Nkx2.5 causing a conformational change that allows binding to the promoter of the atrial natriuretic factor (ANF) which in turn stimulates alternative pathways to promote cardiomyogenesis (Durocher et al., 1997). The activated transcription factor Nkx2.5 also associates with Tbx5 to bind with natriuretic peptide precursor type A (Nppa) to further stimulate the generation of cardiac cells (Hiroi et al., 2001; Stennard and Harvey, 2005).

Given that the acetylation of histone lysine residues by histone acetyltransferases (HATs) increases the accessibility of chromatin DNA for transcription factor binding (Wolffe, 1997; Chen and Li, 2011), it is unsurprising that the p300 that contains an intrinsic HAT activity also plays a major role in cardiac development. By acting as a transcriptional coactivator of GATA4, p300 increases the DNA binding capacity and stability of GATA4 (Dai and Markham, 2001; Kawamura et al., 2005). Genetic evidence suggests that the primary reason for lethality in homozygous p300 knockout mice stems from cardiac defects (Yao et al., 1998). Spatiotemporal analysis of p300 expression demonstrates widespread prevalence in the cardiac crescent and highlights a crucial role in the spatial organization of the developing heart (Chen et al., 2009).

Interestingly, MEF2C also interacts directly with p300 and class II histone deacetylases (HDACs) in a mutually exclusive manner on the same MEF2 domain (Sartorelli et al., 1997; Slepak et al., 2001; Han et al., 2005). Consequently, when class II HDACs are exported out of the nucleus in response to hypertrophic signals, MEF2C interacts with p300 to become fully transcriptionally active and thereby activates downstream genes harboring MEF2C binding sites. These studies strongly suggest that p300 may play an important role in progenitor cell commitment and developmental morphogenesis within the fetal and hypertrophic cardiac development program.

For over four decades, the anticonvulsant drug valproic acid has been used worldwide in the treatment of epilepsy, migraine pain, seizures, and bipolar disorder (Loscher, 1999; Johannessen and Johannessen, 2003). Later, it was identified as a potent HDAC inhibitor. Pertinent to this report, the marked capacity of valproic acid to modulate chromatin and transcription has been exploited to accelerate the induction of pluripotent stem cells from primary human fibroblasts (Phiel et al., 2001; Kramer et al., 2003; Huangfu et al., 2008). In this study, we examined the effect of valproic acid on the specification of cardiac lineage and show that targeting the HDAC activity within pluripotent stem cells enhances cardiac differentiation. We also demonstrate that the direct involvement of histone acetylation and p300 regulate GATA4 gene expression at specific stages of cardiac differentiation, highlighting the precise timing to enable the rational engineering of a ready cell source for cardiac repair.

## Results

### Small molecule inducers in cardiac differentiation

**Figure 1:**
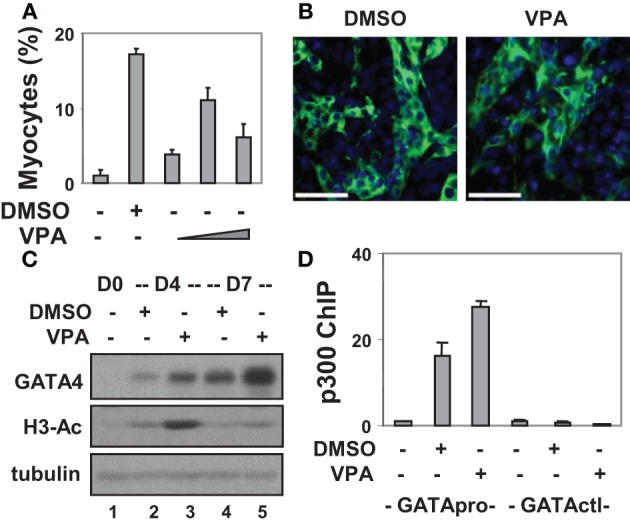
**Valproic acid-enhanced cardiac differentiation. (A)** P19 stem cells were treated with DMSO or increasing concentrations of valproic acid (VPA 0.5, 1, 2 mM) during EB formation, maintained in the tissue culture dishes for 3 additional days without treatments, and then stained for myosin heavy chain and cTnT. Quantification is presented as fractions of cells differentiated into cardiomyocytes relative to the total cell populations. Error bars represent the standard deviations of three independent experiments. **(B)** Shown are the representative microscopy images of the cells stained for cTnT (green). Hoechst was used to stain the DNA (blue) concomitantly (scale bars = 50 μm). **(C)** Western analysis of GATA4 protein expression and the levels of global H3 acetylation. The blots were then stripped and reprobed for β-tubulin as loading controls. Undifferentiated cells were included as a negative control. Shown are the cropped blot images representing indicated protein. **(D)** Occupancy of p300 at the GATA4 promoter (GATApro) and a control locus (GATActl) were examined by a real-time PCR based ChIP analysis. Quantification is presented as the fold variations of undifferentiated control.

To study the molecular regulation of cellular signaling and chromatin modification on cardiac differentiation, we used the P19 pluripotent stem cell model in our initial studies. Previous studies have reported that treatment with 0.5–1% dimethylsulfoxide (DMSO) induces the differentiation of P19 cells into cardiac myocytes (Skerjanc, 1999). Consistent with these reports, 0.8% DMSO converted about 18% of cells into cardiomyocytes as shown by quantitative microscopic analysis of the expression of cardiac troponin T (cTnT) and myosin heavy chain (Figures 1A,B).

To delineate the role of histone acetylation in cardiomyogenesis, we also targeted HDAC activity with an inhibitor approach as a means of increasing histone acetylation. To this end, valproic acid was used and different concentrations were tested for its capacity to induce cardiac differentiation. As shown in Figures 1A,B, about 11% of cells differentiated into cardiomyocytes following valproic acid treatment similar to DMSO induction. In addition, valproic acid enhanced GATA4 gene expression as revealed by the Western analysis (Figure 1C). These findings are consistent with previous reports that HDAC over-expression inhibits the development of cardiomyocytes by down-regulating GATA4 and Nkx2.5 gene expression (Kawamura et al., 2005; Karamboulas et al., 2006; Liu et al., 2009).

Interestingly, both DMSO and valproic acid also increased the level of global histone H3 acetylation as revealed by the Western analysis (Figure 1C). More importantly, they increased the association of transcriptional coactivator p300 to the GATA4 promoter when compared to the undifferentiated cells as shown by a real-time PCR based chromatin immunoprecipitation (ChIP) analysis (Figure 1D). Thus, enhancing histone acetylation and activating gene expression by targeting HDAC activities represent promising course of modulating stem cell fate decisions.

### HAT activity and cardiac differentiation

To determine the molecular basis for p300 involvement in early cardiac differentiation, we used curcumin, a cell permeable compound from *curcuma longa rhizome*, which inhibits the HAT activity of p300 (Balasubramanyam et al., 2004). We have previously optimized the concentrations of curcumin treatments in P19 cell differentiation (Francetic et al., 2012; Hamed et al., 2013) and used 10 μM curcumin in cardiac differentiation since it does not exhibit apparent toxicity to the differentiating cells, while still inhibiting about 40% of p300 HAT activity as determined by a HAT assay (Francetic et al., 2012).

**Figure 2:**
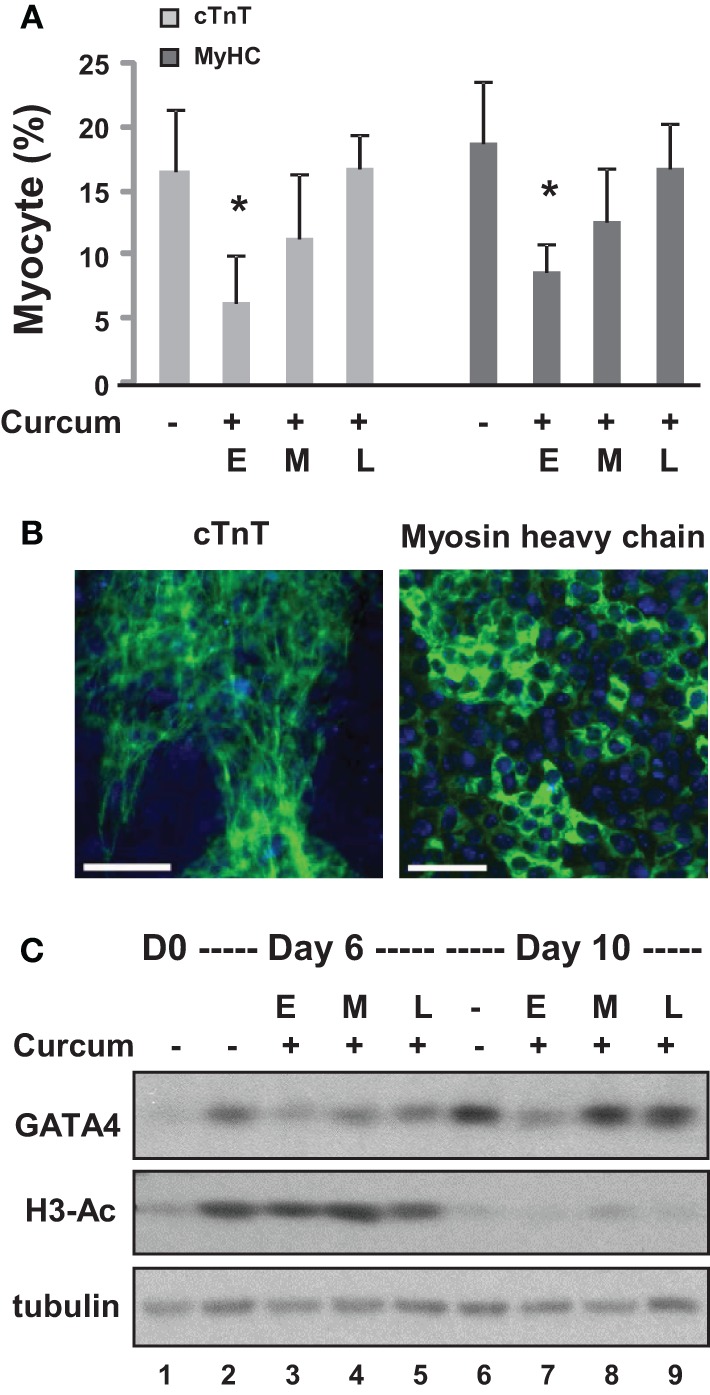
**Effects of curcumin on the differentiation of ES cells into cardiomyocytes. (A)** ES cells were grown in hanging drops for 2 days and in suspension for 4 additional days to form the EBs. Treatment with curcumin (10 μM) was for days 0–2 (E), days 2–4 (M), or days 4–6 (L). The cells were then maintained on cover slips for another 4 days to allow for the development of cardiomyocytes. Quantification is presented as the percentage of cells positive for cTnT and myosin heavy chain (MyHC), respectively. Error bars represent the standard deviations of three independent experiments (^*^p < 0.05). **(B)** Shown are the representative images of untreated cells stained for cTnT or myosin heavy chain (green) on day 10 of differentiation. Hoechst was used to stain the DNA (blue) concomitantly (scale bars = 50 μm). **(C)** Western analysis of GATA4 protein expression and the levels of global H3 acetylation. The blots were then stripped and reprobed for β-tubulin as loading controls. Undifferentiated ES cells and were used as the negative control. Shown are the cropped blot images representing indicated protein.

We first grew the ES cells as hanging drops for 2 days and as suspension culture for 4 additional days to form embryoid bodies (EBs). The EBs were then maintained on coverslips for 4 days during cardiomyocyte development. Curcumin was administered either during the early (day 0–2), middle (day 2–4), or late (day 4–6) stage of EB formation. Quantitative microscopic analysis of cTnT and myosin heavy chain staining demonstrated that curcumin treatment in the early stage led to a significant reduction in cardiac differentiation, by about 60% (Figures 2A,B). However, the inhibitory effect of curcumin progressively reduced when it was administered in later stages of EB formation (Figures 2A,B). The expression of GATA4 was similarly impaired by early curcumin treatment and appeared to associate with a decrease in global histone H3 acetylation (Figure 2C). Treatment in later stage of differentiation did not negatively influence the levels of GATA 4 protein by day 10 of differentiation (Figure 2C).

**Figure 3:**
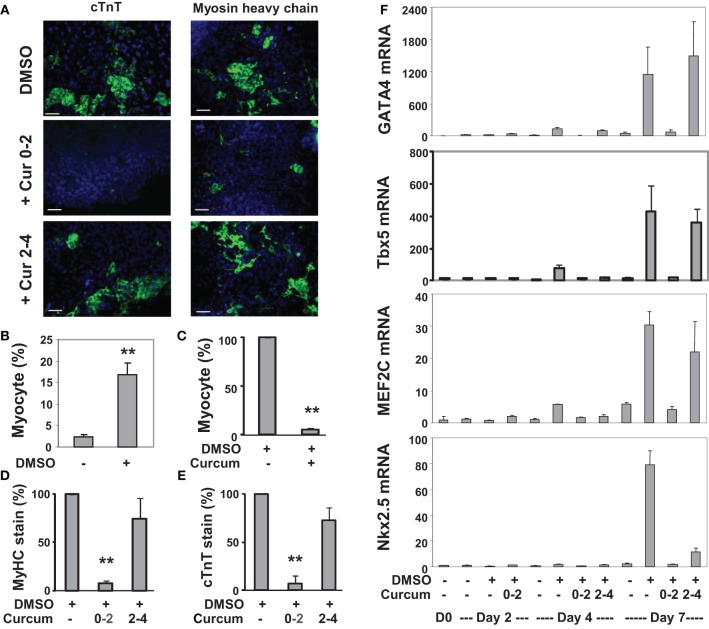
**Effects of curcumin on the differentiation of P19 cells into cardiomyocytes. (A)** P19 stem cells were treated with DMSO during EB formation. The addition of curcumin (10 μM) was during the early stage (days 0–2) or the late stage (days 2–4) of EB formation. The cells were then maintained in the tissue culture dishes for 3 additional days without any treatments, and stained for myosin heavy chain or cTnT (green), and with Hoeschst for the nuclei (blue) (scale bars = 50 μm). **(B)** Quantification of the myosin heavy chain positive cells is expressed as fractions of cardiomyocytes relative to the total cell populations. Error bars are the standard deviations of five independent experiments (^**^p < 0.001). **(C)** Cells were treated with curcumin during EB formation and quantified as the percentage of cardiomyocytes relative to the DMSO control which is defined as 100%. Error bars are the standard deviations of three independent experiments. **(D,E)** The administration of curcumin was during the early stage (days 0–2) or the late stage (days 2–4) of EB formation. Quantification of the percentage of the myosin heavy chain (MyHC) or cTnT positive cells is presented in relation to the DMSO-alone control which is defined as 100%. Error bars are the standard deviations of three independent experiments. **(F)** The mRNA levels of GATA4, Tbx5, MEF2C, and Nkx2.5 were analyzed by real-time RT-PCR using the same batch of cDNA, with GAPDH as internal controls. Quantification is plotted as fold variations of the undifferentiated controls. Error bars represent the standard deviations of the triplicates from one representative experiment.

We also applied curcumin to the P19 differentiation system. Treatment with curcumin during P19 EB formation inhibited about 95% of DMSO-induced cardiomyogenesis (Figures 3A–C). Early curcumin treatment (days 0–2) inhibited about 94% of cardiomyogenesis, whereas the late treatment (day 2–4) inhibited about 25% (Figures 3D,E). GATA4 and Tbx5 transcripts increased about 130- and 75-fold respectively on day 4 following DMSO induction, while MEF2C and Nkx2.5 transcripts increased about 30- and 80-fold respectively on day 7 (Figure 3F). Early curcumin treatment prevented DMSO-enhanced GATA4, Tbx5, MEF2C, and Nkx2.5 transcripts (Figure 3F). However, administration of curcumin in later stage did not affect the levels of GATA4, Tbx5, and MEF2C transcripts, but decreased the Nkx2.5 transcripts, by about 85%, on day 7 (Figure 3F).

**Figure 4:**
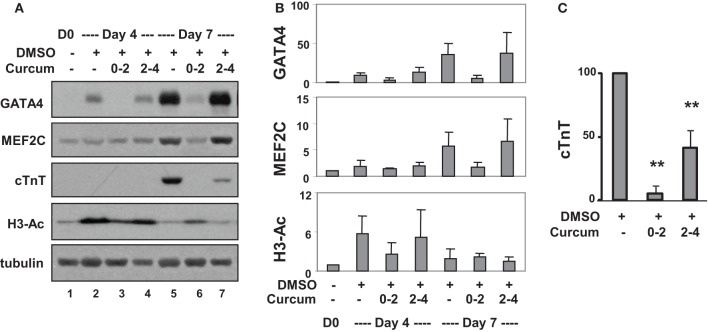
**Effects of curcumin on cardiac protein expression. (A)** P19 stem cells were treated with DMSO and the addition of curcumin (10 μM) was during the early (days 0–2) or the late stage (days 2–4) of EB formation. Western analysis was used to examine the levels of GATA4, MEF2C, and cTnT protein, and global H3 acetylation. The blots were then stripped and reprobed for β-tubulin as loading controls. Undifferentiated cells were used as the negative control. Shown are the cropped blot images representing indicated protein. **(B)** Quantification of the Western blots is expressed as fold variations in relation to the undifferentiated controls (mean ±*SD*, *n* = 3). **(C)** Quantification of cTnT blots is plotted as percentages of the DMSO control (mean ±*SD*, *n* = 3, ^**^p < 0.005 relative to the DMSO control).

Western analysis confirmed that levels of GATA4 protein increased about 10- and 40-fold on days 4 and 7, respectively, compared to undifferentiated cells (Figures 4A,B). Curcumin treatment during day 0–2, but not day 2–4, attenuated GATA4 protein expression, suggesting the activation of GATA4 locus occurs in the early stage of EB formation. MEF2C protein increased about 5-fold on day 7 in DMSO-treated cells relative to the undifferentiated controls (Figures 4A,B). Interestingly, this MEF2C up-regulation was also attenuated by the addition of curcumin during day 0–2, but not day 2–4, suggesting that either the activation of the MEF2C locus occurs in early stage of EB formation, or MEF2C protein expression critically depends on cardiac muscle regulator GATA4 or the expression of unidentified curcumin-sensitive factors. Similar to GATA4 and MEF2C, cTnT gene expression was attenuated by treatment with curcumin during day 0–2 (Figures 4A,C). However, the expression of cTnT protein was also significantly reduced (by about 60%) following curcumin treatment in the later stage (day 2–4), while GATA4 and MEF2C were not affected (Figures 4A,C). Again, global H3 acetylation increased following DMSO induction but reduced following the addition of curcumin (Figures 4A,B).

Taken together, our data demonstrate that curcumin inhibits the specification of cardiac lineage and the expression of cardiac muscle regulators if administered in the early stages of cardiac differentiation. Interestingly, curcumin treatment has little effect on cardiac differentiation when administered after initiation of gene cascades responsible for dictating a cardiac fate. These studies highlight the importance of timely activation of GATA4 gene expression in mesoderm formation for cardiomyogenesis.

### Histone acetylation and p300 occupancy at the GATA4 promoter

**Figure 5:**
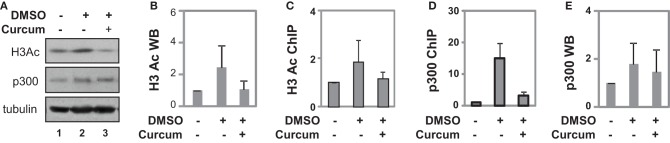
**Occupancy of p300 at the GATA4 promoter at early stage of differentiation. (A)** P19 cells were differentiated with DMSO and co-treatment of curcumin (10 μM) was during the first 2 days of EB formation. The cellular levels of H3 acetylation and p300 protein were analyzed by Western blotting on day 4. The blots were then stripped and reprobed for β-tubulin as loading controls. Undifferentiated cells were used as the negative control. Shown are the cropped blot images representing indicated protein. **(B)** Quantification of acetylated H3 blots is presented as fold variations of the undifferentiated control (mean ± *SD*, *n* = 3). **(C)** The levels of acetylated H3 at the GATA4 promoter were determined by the ChIP analysis. Quantification is presented as fold variations of the undifferentiated control. **(D)** Occupancy of p300 at the GATA4 promoter was examined in parallel. **(E)** Quantification of the p300 Western blots is presented as fold variations of the undifferentiated controls (mean ± *SD*, *n* = 3).

We next determined the effects of curcumin on histone H3 acetylation during P19 cell differentiation to dissect its effects on the GATA 4 promoter from known anti-inflammatory, anti-oxidant, and anti-tumor properties (Balasubramanyam et al., 2004; Chen et al., 2007). Cells were differentiated with DMSO and cotreated with curcumin during the first 2 days of EB formation. As shown by Western analysis, DMSO treatment increased the cellular level of H3 acetylation by about 2-fold compared to the undifferentiated control (Figures 5A,B). The addition of curcumin decreased the DMSO-enhanced H3 acetylation by about 60% (Figures 5A,B).

ChIP analysis revealed that DMSO treatment augmented H3 acetylation at the GATA4 promoter by about 2-fold when compared to undifferentiated cells, while curcumin co-treatment prevented the DMSO-augmented H3 acetylation at the promoter (Figure 5C). While DMSO enriched p300 occupancy at the GATA4 promoter, early curcumin treatment diminished the enrichment of p300 at the promoter (Figure 5D). However, despite the occupancy of p300 being largely reduced at the GATA4 promoter following curcumin treatment, the level of cellular p300 protein was not affected by the treatment (Figures 5A,E). Thus, histone acetylation and p300 association are important for the early activation of GATA4 gene expression at the early stages of cardiac differentiation.

## Discussion

**Figure 6:**
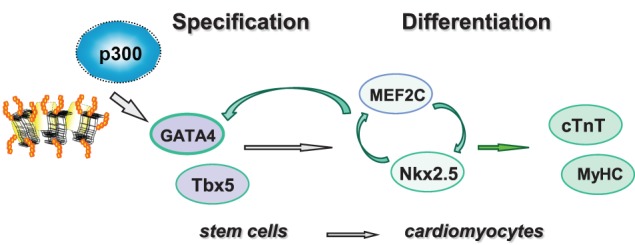
**Shown is the schematic presentation of sequential activation of cardiac factors during the differentiation of stem cells into cardiomyocytes**. Pathway involved in linage specification step is denoted with open gray arrows, whereas pathways involved in the differentiation step are denoted with open green arrows.

In this study, we used targeted small molecules to examine the roles of p300 and histone acetylation in the regulation of GATA4 gene expression. We found that p300 is directly involved in GATA4 gene regulation at the early stage of cardiac specification and that valproic acid is an effective inducer to enhance histone acetylation and cardiac specification (Figure 6).

DMSO is known to induce cardiomyogenesis in P19 stem cells (Skerjanc, 1999), however, the exact mechanism of its action remains largely unknown. It is thought that DMSO induces cardiac differentiation by triggering the release of intracellular calcium stores without phosphoinositide breakdown (Morley and Whitfield, 1993). Other studies have indicated the importance of the Cx43 gap junctions and the oxytocin receptor system in mediating the effects of DMSO (Jasmin et al., 2010). We found that DMSO-enhanced cardiac differentiation was associated with an increase in histone acetylation and a concomitant enrichment of p300 at the GATA4 promoter (Figure 1). Thus, our data indicates a potential mechanism involving histone acetylation and p300-dependent promoter activation in DMSO-mediated cardiac differentiation.

Valproic acid is a popular clinical drug used to treat epilepsy, migraine pain, seizures, and bipolar disorder (Loscher, 1999; Johannessen and Johannessen, 2003). However, its exact mechanism of action has yet to be identified. Studies have shown that it inhibits the activity of class I HDACs and induces the degradation of class II HDACs (along with p300), thereby affecting gene expression (Phiel et al., 2001; Kramer et al., 2003; Chen et al., 2005). In this report, we demonstrate that valproic acid induces cardiac differentiation and GATA4 gene expression which coincides with p300 enrichments at the GATA4 promoter similar to DMSO induction (Figure 1). From a mechanistic point, our data highlights the importance of histone acetylation in cardiac specification.

While the enrichment of p300 at the GATA4 promoter and subsequent GATA4 gene expression is enhanced to a greater degree by valproic acid than DMSO (Figures 1C,D), the ability of DMSO to promote cardiac differentiation is greater than that of valproic acid (Figure 1A). This contrast could be related to the fundamental differences between these inducers in modulating the signaling pathways which may have differential impact on cardiac gene program downstream of GATA4. Nevertheless, treating ES and various induced pluripotent stem cells derived from murine fibroblasts with valproic acid increases the efficiency of cardiomyogenesis regardless of the cell type by increasing the expression of Nkx2.5, ANF, and cardiac contractile proteins (Kaichi et al., 2010).

Curcumin inhibits p300 HAT activity (Balasubramanyam et al., 2004). This inhibitory effect is mediated by its binding to a non-active site region of p300 which results in a conformational change and thereby decreases the binding affinities of p300 to acetylCoA (uncompetitive inhibition) and histones (competitive inhibition). This process takes place by a Micheal reaction, with the α and β unsaturated carbonyl side chains of curcumin serving as the acceptors by forming a covalent bond with p300 (Marcu et al., 2006). The concentration used in our studies is the concentration with effective inhibition of histone acetylation but with minimal cell toxicity (Francetic et al., 2012; Hamed et al., 2013) and we show that HAT activity in the early commitment stage is crucial for cardiac differentiation of ES and P19 cells (Figures 2–4).

The temporal influences of HAT activity were observed in both ES and P19 cardiac differentiation. Early HAT inhibition impeded gene expression of GATA4, MEF2C, TBx5, and Nkx2.5 (Figures 3, 4). These findings suggest that the MEF2C locus may be activated during the early stage of cardiac commitment or that its activation depends on GATA4 or other factors affected directly or indirectly by the HAT activity. The sequential activation cardiac factors during cardiac differentiation is well documented (Figure 6). It is known that MEF2C is a cofactor of GATA4 and plays a role in cardiac, skeletal, and smooth muscle differentiation (Morin et al., 2000). GATA4 also regulates the expression of the Nkx2.5 by activation of its enhancer in combination with SMAD protein (Akazawa and Komuro, 2005). Nonetheless, HAT inhibition in the late stage of differentiation does not affect GATA4, Tbx5, and MEF2C activation, despite Nkx2.5 gene expression being markedly reduced (Figure 3). Taken together, our data indicate that HAT has a temporal involvement in the early commitment stage to the cardiac lineage, in the activation of cardiac muscle regulators and in the synthesis of filament proteins at the later stages of differentiation.

We attempted to elucidate the molecular mechanisms underlying the early commitment to the cardiac lineage by investigating the association of p300 to the GATA4 promoter as well as histone acetylation. Following induction with DMSO, p300 association and H3 acetylation increased at the GATA4 promoter (Figures 5C,D). HAT inhibition impedes the association of p300 to the GATA4 promoter correlating with a decrease in H3 acetylation (Figures 5C,D). Our findings indicate that the HAT activity is important for early H3 acetylation and the recruitment of p300 to the GATA4 promoter, in turn influencing the expression of the GATA4 gene.

It is known that the proximal GATA4 promoter harbors an Nkx2.5 binding site which is important for GATA4 gene expression at the early stages of cardiomyogenesis (Riazi et al., 2009). A recent genome-wide study of cardiac transcription markers has shown that p300 interacts with Nkx2.5 and SRF, and that GATA4 coprecipitates with Nkx2.5 (He et al., 2011). Therefore, it is possible that p300 is recruited to the GATA4 promoter by Nkx2.5 to influence the expression of GATA4, the downstream cascade of the cardiac regulators, and the eventual synthesis of filament proteins at the later stages of cardiac differentiation.

Aside from the role of p300 in normal embryonic development, HAT upregulation is also associated with hypertrophy by reactivating and facilitating the fetal gene program in the adult heart. Transgene-mediated p300 expression in the adult mouse heart has been shown to result in hypertrophy and heart failure (Yanazume et al., 2003). The p300 acetylates regulators such as GATA4, Nkx2.5, and MADS box protein, thereby enhancing their activity to facilitate the activation of the cardiac gene program (Sartorelli et al., 1997; Kakita et al., 1999; Dai and Markham, 2001; Slepak et al., 2001). Curcumin treatment impedes the acetylation of GATA4 by p300 to hinder their interaction and disrupts the downstream fetal gene program in adult murine cardiac tissue subjected to hypertensive and hypoxic injury to significantly improve heart failure outcomes (Morimoto et al., 2008). The current work builds on these studies to indicate the critical time-dependent role that p300 plays in cardiomyogenesis which may have important implications in the activated fetal gene program within stressed adult cardiac tissue.

## Materials and methods

### Cell culture and differentiation

P19 cells (ATCC) were maintained in Minimum Essential Medium α (Invitrogen) supplemented with 5% fetal bovine serum and 5% donor bovine serum (Multicell Wisent) at 37°C with 5% CO2. Differentiation was initiated by growing the cells in Petri dishes in the presence of 0.8% of DMSO (Sigma-Aldrich) or different concentrations of valproic acid (Sigma-Aldrich), dissolved in autoclaved H_2_O. The EBs were then cultured in tissue culture dishes for 3 days. ES cells (ATCC) were maintained in the undifferentiated stage in Dulbecco's Modified Eagle Medium (Invitrogen) supplemented with 15% fetal bovine serum (Multicell Wisent), 1% non-essential amino acids (Gibco), β-mercaptoethanol and Leukemia Inhibitory Factor (LIF, Chemicon). For differentiation, the cells were grown in the absence of LIF as hanging drops for 2 days, in suspension for 4 additional days, and then cultured in tissue culture dishes. Curcumin was purchased from Sigma and dissolved in ethanol for use in treatments.

### Immunofluorescence microscopy

Cells were differentiated as indicated. After attaching to 0.1% gelatin coated coverslips, the cells were fixed with ice cold methanol, air-dried at room temperature and rehydrated in PBS. The cells were then incubated with primary antibody at 4°C overnight, with the secondary antibody Alexa Flor® 488 (Invitrogen) for 2 h, and with 0.1 μg/ml Hoechst (Molecular Probes) for 5 min for nuclear staining. The cells were washed with PBS in between each incubation, and washed before being set on the slides with 10% glycerol(Chen et al., 2004). Following the staining, the coverslips were visualized using Axiovert 200M microscope (Zeiss), AxioCam HRM camera (Zeiss) and AxioVision Rel 4.6 software (Zeiss) (St-Germain et al., 2008). For each coverslip, about 100 fields of view were analyzed and the efficacies of differentiation were estimated based on cells positively stained for cardiac markers cTnT or myosin heavy chain in relation to the total cell population determined by nuclear Hoechst staining. Student *t*-tests were used for statistical analysis. Antibody for myosin heavy chain was from MF20 hybridoma and antibody for cTnT was from Abcam.

### Western analysis

The cells were lysed in whole cell extract buffer (10 % glycerol, 50 mM Tris-HCl pH 7.6, 400 mM NaCl, 5 mM EDTA, 1 mM DTT, 1 mM PMSF, 1 % NP-40). Protein concentration was determined by using a Bio-Rad Protein Assay Dye Reagent (Bio-Rad) and Multiscan Spectrum photospectrometer (Thermo). Equal amounts of protein were separated on SDS-polyacrylamide gel and transferred onto Immun-Blot PVDF membrane (Bio-Rad), probed with specific antibodies and visualized using Western Lightning™ Chemiluminescence reagents (Hamed et al., 2013). The protein bands were quantified using Scion Image software (Scion Corporation). The antibodies for p300, GATA4, and H3Ac were from Santa Cruz Biotechnology. β-tubulin antibody was described previously (Le May et al., 2011).

### Real-time RT-PCR analysis

The total RNA was isolated by using RNeasy Mini kit (Qiagen). Multiskan Spectrum spectrophotometer (Thermo) was used to determine the concentrations and the quality of the RNA (Chen et al., 2010). Equal amounts of RNA were used in reverse transcription with High Capacity cDNA Archive Kit—Multiscribe Reverse Transcriptase (Applied Biosystems). Quantitative real-time PCR amplification was carried out by using the Applied Biosystems 7500 Fast Real-Time PCR System. The amount of targets, normalized to the GAPDH endogenous reference and relative to calibrator control is calculated using the arithmetic formula 2^−ΔΔCT^. The GATA4, Nkx2.5 and Tbx5 primer have been described previously (Li et al., 2013). The primers for MEF2C are the following:

MEF2C fwd- TCAGTTGGGAGACCGTACCAC

MEF2C rev- CCATCGTAGGAACTGCTACAG C

### Chromatin immunoprecipitation

The P19 EBs were crosslinked with 1% formaldehyde and lysed using ChIP Lysis Buffer (50 mM Tris-HCl pH 8.0, 10 mM EDTA pH 8.0, 1%SDS, 1X protease inhibitors, 1 mM DTT, 1 mM PMSF), and then sonicated with the Bioruptor system (Diagenode). The required amount of chromatin was diluted in ChIP Dilution Buffer (20 mM Tris-HCl pH8.0, 150 mM NaCl, 2 mM EDTA, 1% Triton X-100, 1x Protease inhibitors) and incubated with the indicated antibodies overnight at 4°C. Anti-IgG serum was used as a negative control. The chromatin-antibody complexes were pulled down by incubation with Dynabeads Protein A (Invitrogen) for 2 h. The beads were then washed and processed as previously described (Le May and Li, 2013). Purified DNA targets were quantified by Real-time PCR. The fold increase was determined with respect to the undifferentiated control, with input chromatin DNA used as an internal control. Antibodies for p300 and acetylated H3 were from Santa Cruz Biotechnology. The GATA4 primers have been described previously (Voronova et al., 2012).

Primers for GATA4 control:

Fwd-GAGCTGGTACCTGGCCTT C

Rev- GCTCTGCTGAAATCACTCTGA

### Conflict of interest statement

The authors declare that the research was conducted in the absence of any commercial or financial relationships that could be construed as a potential conflict of interest.
